# Love-Wave Sensors Combined with Microfluidics for Fast Detection of Biological Warfare Agents

**DOI:** 10.3390/s140712658

**Published:** 2014-07-15

**Authors:** Daniel Matatagui, José Luis Fontecha, María Jesús Fernández, Isabel Gràcia, Carles Cané, José Pedro Santos, María Carmen Horrillo

**Affiliations:** 1 Instituto de Tecnologías Físicas y de la Información (ITEFI), CSIC, Serrano 144, Madrid 28006, Spain; E-Mails: joseluis.fontecha@csic.es (J.L.F.); mj.fernandez@csic.es (M.J.F.); jp.santos@csic.es (J.P.S.); 2 Centro de Ciencias Aplicadas y Desarrollo Tecnológico (CCADET), Universidad Nacional Autónoma de México (UNAM), 04510 Distrito Federal, Mexico; E-Mail: daniel.matatagui@ccadet.unam.mx; 3 Instituto de Microelectrónica de Barcelona, CSIC, Campus UAB, Bellaterra 08193, Spain; E-Mails: isabel.gracia@csic.es (I.G.); carles.cane@imb-cnm.csic.es (C.C.)

**Keywords:** biological warfare agent, BWA, love-wave, sensor acoustic wave, SAW, biosensor, immunosensor, microfluidics, bacteriophage

## Abstract

The following paper examines a time-efficient method for detecting biological warfare agents (BWAs). The method is based on a system of a Love-wave immunosensor combined with a microfluidic chip which detects BWA samples in a dynamic mode. In this way a continuous flow-through of the sample is created, promoting the reaction between antigen and antibody and allowing a fast detection of the BWAs. In order to prove this method, static and dynamic modes have been simulated and different concentrations of BWA simulants have been tested with two immunoreactions: phage M13 has been detected using the mouse monoclonal antibody anti-M13 (AM13), and the rabbit immunoglobulin (Rabbit IgG) has been detected using the polyclonal antibody goat anti-rabbit (GAR). Finally, different concentrations of each BWA simulants have been detected with a fast response time and a desirable level of discrimination among them has been achieved.

## 1. Introduction

An ideal Biological Warfare Agent (BWA) or bioagent is a microorganism or toxin with a high ability to infect or intoxicate with small doses and easy to disperse. It is also characterised by the following attributes: high virulence or lethality, short incubation period, symptoms that appear in a short time, no widespread immunity, resistance to treatments with common medications, easily stored, transported and propagated, strong ability to survive extreme environmental conditions, and the possibility to reproduce it in high volumes with a small laboratory infrastructure and to be perfected through genetic engineering. Therefore, there is an increasing interest in the development of a system that detects BWAs (bacteria, viruses or toxins) in a short time and with efficient identification, hence determining the source of infection (food, water, and air) and stopping the spread of diseases.

The standard laboratory diagnostic tests dedicated to detecting bacteria, viruses or toxins are based on culture in media such as double-layer agar (DLA), which is highly sensitive, but the procedure is cumbersome and time-consuming. Another alternative is the polymerase chain reaction (PCR) technique, a popular method which can be run in 30 min; however it requires very sterile laboratories and an extremely well-trained staff. A laborious pre-processing of the analyte is necessary for other conventional analytical methods such as high-performance liquid chromatography (HPLC) or Enzyme-Linked ImmunoSorbent Assay (ELISA). Due to the mentioned drawbacks, the development of a system to detect BWAs in real-time and *in situ* is urgently needed.

Nowadays, a great effort to develop miniaturised systems that integrate multiple laboratory functions into a single chip is being realised, thus replacing standard laboratory diagnostics. These systems, known as “lab on a chip,” represent the most promising alternative in detecting BWAs in real time and *in situ*. Most of the latter systems used to detect bioagents are based on biosensors [1–8] joined with microfluidics [9–11]. Among acoustic wave (AW) sensors [6,12–15], Love wave devices are very promising because they show the highest mass sensitivity when working in liquid media. It is well known that a Love wave has a pure shear horizontal polarisation that suffers low attenuation when working with liquid media [16–25]. A technique to detect a BWA by means of solid state sensor is based on immunoreactions of antibody-antigen binding recognition (also called “immunosensors”) generally rely on highly sensitive devices to translate the biological recognition in a physical magnitude variation in real time. On the other hand, the use of microfluidics in the field of medicine and security is increasingly common due to the advantages that it provides: small sample volumes, unnecessary expensive reagents, easy fluid control by the use of pumps, and the ease of automating the detection when dynamic mode is used. Furthermore, the Love wave device and the microfluidics chip are easily combinable [22,23,26].

Though several papers about immunosensors based on Love-wave devices have been published in the past, this work specifically demonstrates the fast detection time and high response rate of the Love-wave sensors combined with microfluidics operating in a dynamic mode, making these systems a powerful tool in detecting BWAs.

## 2. Experimental Section

### 2.1. Immunoassays

Biological warfare agents are very dangerous for human health, and for security reasons are only measured in special installations. Therefore, BWA simulants (non-pathogenic for humans) were used instead of these ones.

- Rabbit immunoglobulin and antibody of goat anti-rabbit

The rabbit immunoglobulin (Rabbit-IgG, I8140, Sigma, St. Louis, MO, USA) was used in order to simulate general BWAs (bacteria, virus or toxin), and it was recognised by means of the polyclonal antibody of goat anti-rabbit (GAR, R2004, Sigma) [6].

- M13 bacteriophage and AM13 antibody

The viruses used as BWAs were simulated by means of the bacteriophage M13 (Stratagene, La Jolla, CA, USA). This one is a filamentous bacteriophage (which infects bacteria) [21,27], with a length of 900 nm, diameter of 9 nm, and a weight of 3.1 × 10^−18^ kg. It is composed of an encapsulated DNA in a coating consisting of 2800 copies of the protein P8 and five copies of the proteins P3, P6, P7 and P9. The protein P3 attaches to the receptor at the tip of the F pilus of the host *Escherichia coli*, infecting the bacteria and causing plaques. Due to the great affinity of the mouse monoclonal antibody anti-M13 (AM13, Amersham-Pharmacia Biotech, Piscataway, NJ, USA) to the protein P8, M13, was used to link the bacteriophage, for the immunoreaction.

### 2.2. Love-Wave Sensor

Love-wave devices with a size of 30 mm × 40 mm × 0.5 mm were used; all of them containing two delay lines (DL). They were based on a shear horizontal surface acoustic wave (SH-SAW) propagated on the ST-cut quartz, perpendicular to the x crystallographic axis. This SH-SAW, with a wavelength of λ = 28 μm was generated and detected by interdigital transducers (IDTs). The IDTS were made using standard lithographic techniques by depositing an aluminum layer with a thickness of 200 nm through RF sputtering. A double electrode structure was repeated 75 times to form each IDT. The spacing, centre to cente, between the IDTs was 225 λ, and the acoustic aperture was 75 λ. The surface area of recognition was 7.4 mm^2^ (the width of the microchannel was 3.4 mm). Finally, the Love wave was obtained by guiding the SH-SAW in a film of SiO_2_ deposited by PECVD. The highest sensitivity was found at a thickness of approximately 3.5 μm of SiO_2_ calculated following the method of Wang [28] and fully described for our case in [29]. The synchronous frequency for this device is around 163 MHz.

The sensors were characterised using a vector network analyser (Agilent 5070B, Englewood, CO, USA) and a spectrum analyser (Agilent 9320A). Then, each DL of the Love device was connected to an amplifier circuit in order to make an oscillator, and each circuit transmitted a small part of the signal energy to a channel where the frequency counter (Agilent 53131A) was connected.

### 2.3. PDMS Chip

**Figure 1.**
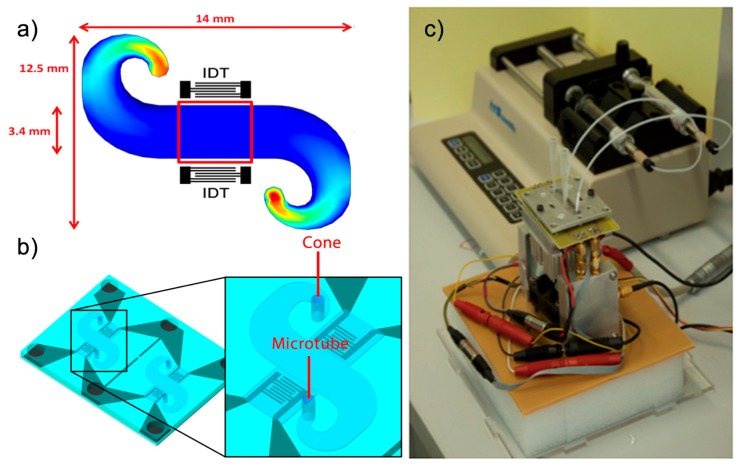
(**a**) Simulation of velocity of the liquid in the microchannel (in red the highest velocity and in blue the lowest one); (**b**) Love-wave device with two delay lines and a microfluidic chip of PDMS forming a microchannel; (**c**) Photography of the liquid cell and syringe pump.

In order to achieve a uniform velocity of the sample in the path between the IDTs, Comsol software was used to simulate the flow of the liquid in a microchannel before the microfluidic system was developed (Figure 1a).

To obtain the microfluidic circuit, a chip of polydimethylsiloxane (PDMS) with a groove in the microchannel simulated was used. Thus, by joining the PDMS chip and the Love-wave device, microchannels were formed. In addition, micro-chambers of air are needed on the IDTs to prevent contact with liquid and the PDMS. Therefore, a wall was created to serve as an insulation between the microchannel and the air chamber that protects the IDTs, keeping both completely isolated. The PDMS is a material that absorbs a large amount of energy from acoustic waves. The following points have been taken into account for the present study:

- The PDMS in contact with the chip will be used as an advantage to weaken the energy that propagates backwards from the IDT generator to the edge of the device.
- Two walls block the path of the acoustic wave between the IDTs, separating the IDT area from the microchannel.

As the walls attenuate the amplitude of the wave and prevent leakage of fluids into the IDT area, it is important to set an appropriate wall dimension. The microchannel has a height of 150 microns; thus walls with the same width were chosen for this study in order to ensure that they do not deform by the pressure exerted.

In order to shape the PDMS, a SU8 mold was used as a master to make the PDMS chip. The microchannel structures and cavities on the IDT area were then defined by means of a photolithographic process [22]. Then, to obtain the PDMS chip the silicone base and curing agent (ELASTOSIL 601 RT, Wacker München, Germany) were mixed in a 9:1 weight ratio. The mixture was degassed to remove any bubble and poured over the master and degassed again, ensuring all entrapped gases were evacuated. After baking and cooling, the PDMS was easily peeled and cut. The microstructure of PDMS and the Love wave device were joined by pressure, thus forming microchannels of 150 μm of height without leakages (Figure 1b).

### 2.4. Experimental Setup

The measurements were based on a continuous flow through the microchannel. The sample was deposited in a cone that was placed on one side of the microchannel. The other side of the microchannel was connected to a syringe pump (210-CE, KDScientific, Holliston, MA, USA) through a microtube. This pump operated in suction mode, producing a constant flow of the liquid which was in the cones, which flowed through the microchannel and eventually reached the syringe through the microtube, in which all the test samples were stored as a residue. The rate of fluid through the microchannel was adjusted by the speed selected in the syringe pump. Love wave sensors are very sensitive at the temperature fluctuations. Therefore, a Peltier device controlled by a PID programme from the computer was used in order to keep constant the temperature at 30 °C, which was measured by a Pt100 sensor.

### 2.5. Modification of the Surface of the Love-Wave Device

In order to link the antibodies, the surface of the device was oxidised and thus activated [6] by depositing fresh piranha etch (H_2_SO_4_:H_2_O_2_, 3:1, v/v), provided by Sigma-Aldrich (Sigma 30743 and Sigma 18312, respectively), on the SiO_2_ surface for 5 min. To continue the activation process, the Love-wave device was immersed in a solution of 20 mM 3-aminopropyl triethoxysilane (APTES) (Sigma A3648) with toluene (Sigma 32249) for 1 h (15 min in sonication, 15 min of rest, 15 min in sonication, and 15 min of rest), followed by a thorough cleaning with toluene and isopropanol (Sigma 33539). The APTES covered the surface with amine-terminated silane, thus the surface was prepared to react with 20 mM of glutaraldehyde (GA) (Sigma 340855, 50 wt. % in H_2_O) solution for one hour. Therefore, GA was used as a homo-biofunctional cross-linker between the amine groups of the APTES and the primary amines of the immunoglobulin.

## 3. Results and Discussion

### 3.1. Simulations of the Detection in Static and Dynamic Modes

**Figure 2.**
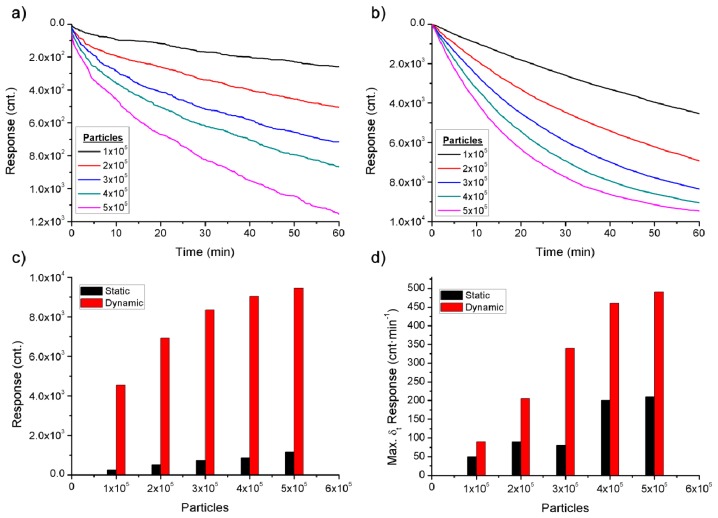
Simulation of (**a**) detection in static mode; (**b**) detection in dynamic mode; (**c**) quantification of the concentration by the absolute value of the sensor response after 60 min; and (**d**) quantification of the concentration taking the maximum value of the sensor response per minute.

The detection of bioagents in static and dynamic modes is controlled by very different processes, for this reason, in this paper the detection in dynamic mode in order to improve the efficiency of the sensor is proposed. The static and dynamic modes were simulated in Matlab software (Figure 2) to corroborate the theory background. The different simulated detections were based on the details of the theory and data found below.

From a physical point of view, the bioagents can be approximated by spherical particles suspended in a liquid. A Brownian movement characterises the bioagents when the fluid is a rest, as was expressed mathematically in the Stocks–Einstein diffusion equation:

(1) $$D=\frac{k_{B}T}{6\pi \mu r}$$

where *k_B_* [J·K^−1^], the Boltzmann constant, *T* [K] the temperature, *r* [m] the sphere radius, and μ [N·s·m^−2^] the dynamic viscosity of the liquid. For example, a bioagent with 100 nm of radius (a virus), mixed with water at a temperature of 30 °C will produce a diffusion of 5.6 × 10^−12^ m^2^·s^−1^. This means that, when the fluid is at rest, the maximum velocity that a virus can approach the surface with antibodies is 2 × 10^−4^ m·h^−1^ (it was taken into account in the simulation), implying that the process of detection occur in two periods when in static mode: first, a rapid process due to immunoreaction of the bioagents close to the antibodies; then, a slow process in which the farther bioagents reach the antibodies by diffusion displacement (Figure 2a). However it is of interest that the maximum number of bioagents reaches the surface quickly and interacts with the identifier element in order to obtain the maximum sensor response in the shortest time. Therefore, the bioagents are carried by the fluid when in dynamic mode, regenerating the concentration of bioagents close to antibodies which is dependent on the velocity of the fluid (velocity of the bioagents at the simulation 0.6 m·h^−1^) (Figure 2b). As such, the displacement velocity of the bioagents is the main difference between the static and dynamic modes. The slow velocity of the bioagents causes a lower response rate of the sensor in static mode, whereas in dynamic mode the higher velocity promotes the immunoreaction over time of the detection. In detection the sensor response is only stable when the immunoreaction is saturated. In fact the sensor response tends to saturation much faster in dynamic mode than for static mode, improving the sensor response but making the quantification of the concentration of bioagents difficult when the immunoreaction is close to saturation, as shown in the Figure 2c. Consequently, taking the maximum value of the sensor response per minute, it is possible to quantify each concentration in a few minutes (Figure 2d). The simulations shown that in static mode (Figure 2a), the response of the sensor is about one order of magnitude lower than in dynamic mode (Figure 2b) and this difference is increased with larger BWAs, due to the slower diffusion (Equation (1)).

### 3.2. Detection of the BWA Simulants

The use of microchannels allowed the Love wave sensor to operate in dynamic mode with an appropriate flow and for an extended time using a few microlitres of sample. In order to obtain an efficient detection system for BWAs and obeying the theory, a system of a Love-wave device combined with microfluidics was developed and used to detect two BWA simulants. After the process of surface modification, the Love-wave device and the PDMS chip were joined and mounted onto the measurement system. The cones were then filled with 200 μL of TBS and a flow of 10 μL·min^−1^ was selected. Once the frequency was stable, the solution of antibodies was mixed with TBS in the cone to obtain a final concentration of 100 μg·mL^−1^ and 50 μg·mL^−1^ for the GAR and AM13 respectively, and was passed through the microchannel where the antibodies were bound to the surface. In order to remove the antibodies remaining in the cone as well as those with a weak bond linked to the surface, a rinsing with TBS was carried out after the antibodies were immobilised.

**Figure 3.**
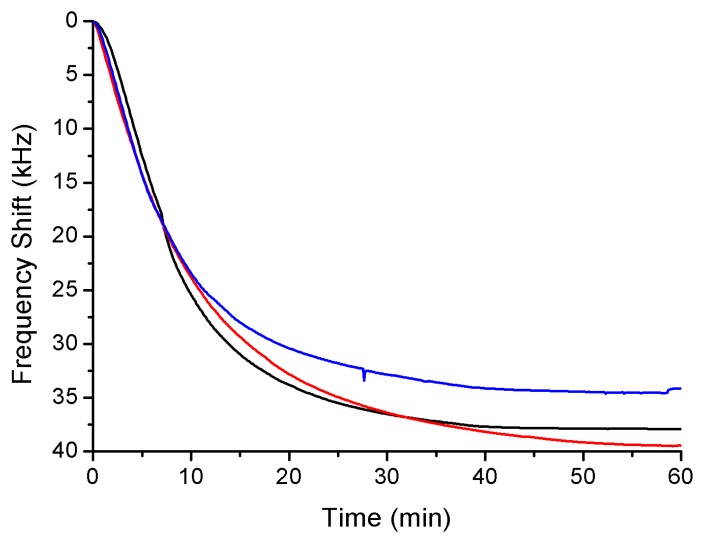
Frequency shift for three different immobilizations of the GAR antibody on the surface of SiO_2_.

The Love device is a mass sensor; thus there is a correlation between the displacement of the resonance frequency and the amount of the bound antibodies, similar frequency shifts indicated a similar number of bound antibodies in the process of detection. Furthermore, there is a relationship between the number of bound antibodies and bioagents detected. In Figure 3, three responses to the GAR antibody are compared, obtaining a displacement of 37 ± 2.5 kHz. Due to the high reproducibility of the binding of antibodies, it was possible to compare different responses of the detection of each BWA simulant.

After immobilisation of the antibodies, the modified surface was blocked with bovine serum albumin (BSA), and then the surface was rinsed. Thus the immunosensor was prepared to recognise the BWA simulant, which was introduced into the cone in the desired concentration. After the detection, the immunosensor was rinsed to verify the detection through immunoreaction. As the bioagent remained captured by the antibody, the frequency was not recovered.

**Figure 4.**
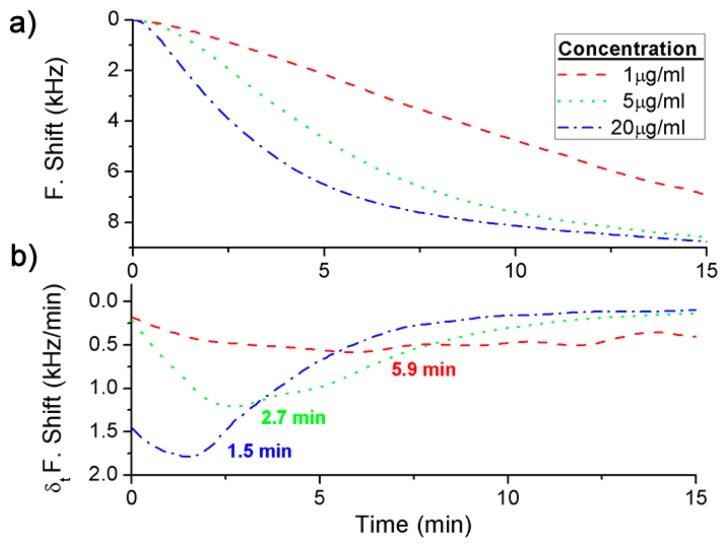
Frequency shift for (**a**) the measurements in real time of the different concentrations of the Rabbit IgG and (**b**) their derivate with respect to time.

**Figure 5.**
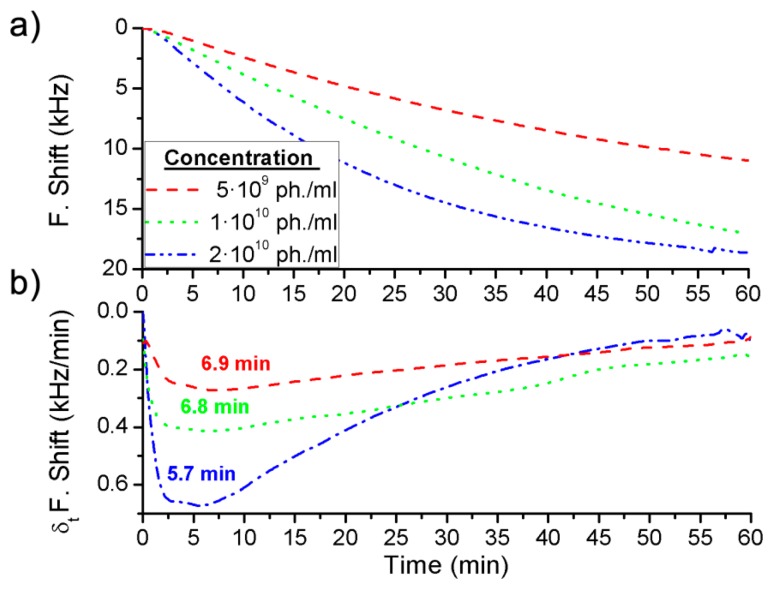
Frequency shift for (**a**) the measurements in real time of the different concentrations of the bacteriophage M13; and (**b**) their derivate with respect to time.

Three different concentrations were detected for each BWA simulant using different immunoassays: 2 μg·mL^−1^, 5 μg·mL^−1^ and 20 μg·mL^−1^ of the Rabbit IgG with a flow rate of 3 μL·min^−1^ (Figure 4a), and 5 × 10^9^ pfu·mL^−1^, 1 × 10^10^ pfu·mL^−1^ and 2 × 10^10^ pfu·mL^−1^ of the bacteriophage M13 with a flow rate of 2 μL·min^−1^ (Figure 5a).

**Figure 6.**
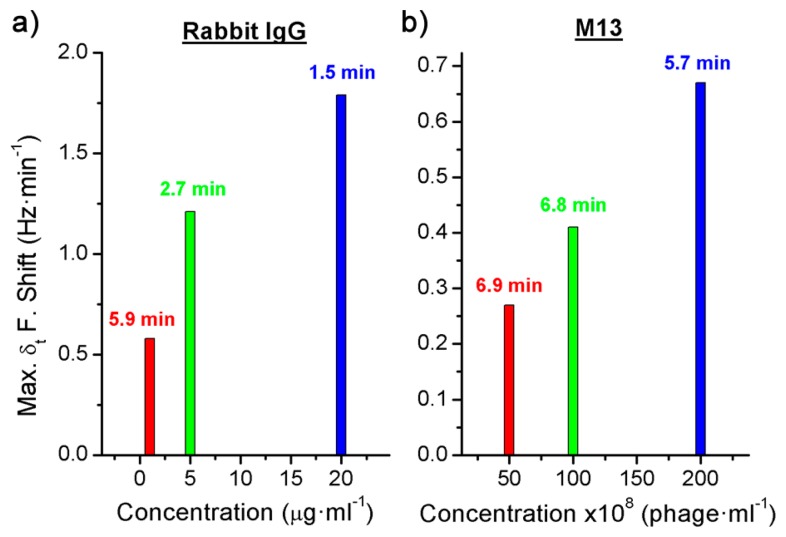
Maximum frequency shift per minute of the different concentrations (**a**) of the Rabbit IgG and (**b**) of the bacteriophage M13.

In addition, the derivate of the frequency with respect to the time was calculated and, as it has been explained above, the maximum value of the frequency shift per minute was used to quantify each concentration (Figures 4b and 5b). The difference between the real case and simulated case was that in the real case there was a delay until the maximum concentration of the bioagent reaches the sensor. Hence the maximum value of the frequency shift per minute was detected by the time derivative of the frequency shift. But this time was short and it was possible to quantify each concentration in a few minutes (Figure 6). On the other hand, Figure 4a shows the saturation was for around 9 kHz, and for the different concentrations of the Rabbit IgG, after 15 min of detecting, the immunoreactions were close to saturation. However, in static mode for lower concentrations (1 μg·mL^−1^ and 5 μg·mL^−1^) and after 1 h the sensor was not saturated [6], therefore the immunoreactions were slower. In the case of the M13 for concentrations higher than 10^10^ pfu·mL^−1^ the immunoreaction is saturated after 1 h. Nevertheless Tamarin *et al.* [21] carried out the detection of M13 in static mode and for higher concentrations than in this work, and after over 1 h of detecting that the immunoreaction was not saturated, it was evident that in dynamic mode the immunoreaction is faster.

## 4. Conclusions

In static mode the movement of bioagents is only due to diffusion. With this slow process the bioagents do not quickly reach the antibodies. Nevertheless, with the dynamic mode the interaction between antibodies and biological warfare agents is promoted, improving the detection time and increasing the response of the sensor.

The simulations realised have shown that the response of the sensor in static mode is about one order of magnitude lower than in dynamic mode and this difference increases with larger BWA, due to the slower rate of diffusion. The difference between the responses is caused by the variation in velocity that dominates the process, the diffusion in static mode and the fluid velocity in dynamic mode.

Two different immunoreactions were performed to validate the detection system developed for biological warfare agents, for this purpose two simulants were used: the Rabbit IgG and the bacteriophage M13, detected by the goat anti-rabbit and the anti-M13 antibodies respectively. Therefore a reusable Love-wave immunosensor combined with microfluidic structures were used in order to work in dynamic mode and use a small volume of sample. This system has shown a fast response for the low concentrations of BWAs simulants detected. In addition, by means of the frequency derivate with respect to the time it has been possible to detect and quantify the BWA simulants in a few minutes.

## References

[1] Gooding J.J. (2006). Biosensor technology for detecting biological warfare agents: Recent progress and future trends. Anal. Chim. Acta.

[2] Richardson J., Hawkins P., Luxton R. (2001). The use of coated paramagnetic particles as a physical label in a magneto-immunoassay. Biosens. Bioelectron..

[3] Shew B.Y., Cheng Y.C., Tsai Y.H. (2008). Monolithic su-8 micro-interferometer for biochemical detections. Sens. Actuators A Phys..

[4] Wildeboer D., Jiang P., Price R.G., Yu S., Jeganathan F., Abuknesha R.A. (2010). Use of antibody-hapten complexes attached to optical sensor surfaces as a substrate for proteases: Real-time biosensing of protease activity. Talanta.

[5] Qi C., Tian X.S., Chen S., Yan J.H., Cao Z., Tian K.G., Gao G.F., Jin G. (2010). Detection of avian influenza virus subtype h5 using a biosensor based on imaging ellipsometry. Biosens. Bioelectron..

[6] Oliver M.J., Hernando-García J., Pobedinskas P., Haenen K., Ríos A., Sánchez-Rojas J.L. (2011). Reusable chromium-coated quartz crystal microbalance for immunosensing. Colloids Surfaces B Biointerfaces.

[7] Dudak F.C., Boyaci I.H. (2014). Peptide-based surface plasmon resonance biosensor for detection of staphylococcal enterotoxin b. Food Anal. Methods.

[8] Weingart O.G., Gao H., Crevoisier F., Heitger F., Avondet M.A., Sigrist H. (2012). A bioanalytical platform for simultaneous detection and quantification of biological toxins. Sensors.

[9] Raimbault V., Rebière D., Dejous C., Guirardel M., Conedera V. (2008). Acoustic love wave platform with pdms microfluidic chip. Sens. Actuators A Phys..

[10] Novo P., Chu V., Conde J.P. (2014). Integrated optical detection of autonomous capillary microfluidic immunoassays: A hand-held point-of-care prototype. Biosens. Bioelectron..

[11] Matatagui D., Moynet D., Fernández M.J., Fontecha J., Esquivel J.P., Gràcia I., Cané C., Déjous C., Rebière D., Santos J.P. (2013). Detection of bacteriophages in dynamic mode using a love-wave immunosensor with microfluidics technology. Sens. Actuators B Chem..

[12] Grate J.W., Martin S.J., White R.M. (1993). Acoustic wave microsensors. Part ii. Anal. Chem..

[13] García-Martinez G., Bustabad E.A., Perrot H., Gabrielli C., Bucur B., Lazerges M., Rose D., Rodriguez-Pardo L., Fariña J., Compère C. (2011). Development of a mass sensitive quartz crystal microbalance (qcm)-based DNA biosensor using a 50 mhz electronic oscillator circuit. Sensors.

[14] Lazerges M., Perrot H., Rabehagasoa N., Compère C. (2012). Thiol- and biotin-labeled probes for oligonucleotide quartz crystal microbalance biosensors of microalga alexandrium minutum. Biosensors.

[15] Fourati N., Lazerges M., Vedrine C., Fougnion J.M., Zerrouki C., Rousseau L., Lepeut P., Bonnet J.J., Pernelle C. (2009). Surface acoustic waves sensor for DNA-biosensor development. Sens. Lett..

[16] Rocha-Gaso M.I., March-Iborra C., Montoya-Baides A., Arnau-Vives A. (2009). Surface generated acoustic wave biosensors for the detection of pathogens: A review. Sensors.

[17] Blondeau-Patissier V., Boireau W., Cavallier B., Lengaigne G., Daniau W., Martin G., Ballandras S. (2007). Integrated love wave device dedicated to biomolecular interactions measurements in aqueous media. Sensors.

[18] Bisoffi M., Hjelle B., Brown D.C., Branch D.W., Edwards T.L., Brozik S.M., Bondu-Hawkins V.S., Larson R.S. (2008). Detection of viral bioagents using a shear horizontal surface acoustic wave biosensor. Biosens. Bioelectron..

[19] Branch D.W., Brozik S.M. (2004). Low-level detection of a bacillus anthracis simulant using love-wave biosensors on 36°YX LiTaO_3_. Biosens. Bioelectron..

[20] Ravula S.K., Branch D.W., James C.D., Townsend R.J., Hill M., Kaduchak G., Ward M., Brener I. (2008). A microfluidic system combining acoustic and dielectrophoretic particle preconcentration and focusing. Sens. Actuators B Chem..

[21] Tamarin O., Comeau S., Déjous C., Moynet D., Rebière D., Bezian J., Pistré J. (2003). Real time device for biosensing: Design of a bacteriophage model using love acoustic waves. Biosens. Bioelectron..

[22] Matatagui D., Fontecha J., Fernández M.J., Oliver M.J., Hernando-García J., Sánchez-Rojas J.L., Gràcia I., Cané C., Santos J.P., Horrillo M.C. (2013). Comparison of two types of acoustic biosensors to detect immunoreactions: Love-wave sensor working in dynamic mode and qcm working in static mode. Sens. Actuators B Chem..

[23] Fournel F., Baco E., Mamani-Matsuda M., Degueil M., Bennetau B., Moynet D., Mossalayi D., Vellutini L., Pillot J.P., Dejous C. (2012). Love wave biosensor for real-time detection of okadaic acid as dsp phycotoxin. Sens. Actuators B Chem..

[24] Melzak K.A., Ellar D.J., Gizeli E. (2004). Interaction of cytolytic toxin cytb with a supported lipid bilayer: Study using an acoustic wave device. Langmuir.

[25] Länge K., Rapp B.E., Rapp M. (2008). Surface acoustic wave biosensors: A review. Anal. Bioanal. Chem..

[26] Fu Y.Q., Luo J.K., Du X.Y., Flewitt A.J., Li Y., Markx G.H., Walton A.J., Milne W.I. (2010). Recent developments on zno films for acoustic wave based bio-sensing and microfluidic applications: A review. Sens. Actuators B Chem..

[27] Makowski L. (1992). Terminating a macromolecular helix. Structural model for the minor proteins of bacteriophage m13. J. Mol. Biol..

[28] Wang Z., Cheeke J.D.N., Jen C.K. (1994). Sensitivity analysis for love mode acoustic gravimetric sensors. Appl. Phys. Lett..

[29] Matatagui D. (2012). Sensores Másicos Para la Detección de Agentes de Guerra Química y Biológica. Ph.D. Thesis.

